# Oxygen is toxic in the cold in *C. elegans*

**DOI:** 10.3389/fphys.2024.1471249

**Published:** 2024-12-24

**Authors:** Cameron M. Suraci, Michael L. Morrison, Mark B. Roth

**Affiliations:** Roth Lab, Basic Sciences, Fred Hutchinson Cancer Research Center, Seattle, WA, United States

**Keywords:** oxygen, temperature, cold acclimatization, *C. elegans*, oxidative stress, cold shock, survival, ROS

## Abstract

**Introduction:**

Temperature and oxygen are two factors that profoundly affect survival limits of animals; too much or too little of either is lethal. However, humans and other animals can exhibit exceptional survival when oxygen and temperature are simultaneously low. This research investigates the role of oxygen in the cold shock death of Caenorhabditis elegans.

**Methods:**

The survival of *C. elegans* populations in combinations of oxygen concentrations and was assayed. Additionally, the effect of cold acclimatization, mutations in the cold acclimatization pathway, compounds, and antioxidant proteins on survival in low temperatures and high oxygen were investigated.

**Results:**

We demonstrate that *C. elegans* have increased survival in 2°C when deprived of oxygen, and an increase to just 0.25 kPa of oxygen decreased survival. Additionally, we show that oxygen toxicity produced by a 35-fold increase above atmospheric oxygen levels was fatal for nematodes in 8 h at room temperature and 2 h at 2°C. We found that cold acclimatization and mutations in the cold acclimatization pathway improve survival in room temperature oxygen toxicity. Furthermore, we found that the compounds glucose, manganese (II), and ascorbate improve both cold shock and high oxygen survival, while the antioxidant proteins catalase and peroxiredoxin are essential to wild type survival in these conditions.

**Discussion:**

Our results suggest that oxygen toxicity contributes to the death of *C. elegans* during cold shock. The changes in survival induced by cold acclimatization and mutations in the cold acclimatization pathway suggest that oxygen toxicity in the cold exerts evolutionary pressure, leading to the development of protections against it. Additionally, the resistance provided by diverse compounds and antioxidant proteins in both low temperature and high oxygen suggests these conditions have similar chemical environments. We discuss evidence that similar phenomena may function in humans.

## 1 Introduction

Prolonged exposure to low oxygen or low temperature is lethal in most animals. Despite this, some humans, such as airplane wheel-well stowaways, have anomalously survived conditions where either the low oxygen alone or the cold alone would have been enough to ensure death (Veronneau et al., 1996). One possible explanation of this anomaly is that low oxygen and low temperature are synergistically protective. The protection the cold provides against the effects of low oxygen has been applied in humans: cooling is used to prevent brain damage in hypoxic infants (Lemyre and Chau, 2018). However, the protection that low oxygen provides against the effects of the cold, or the potential damaging effects of high oxygen at these low temperatures, are less understood and have not been applied in therapeutic settings.

To understand more about the relationship between oxygen and temperature we have been studying the nematode *C. elegans*. This animal has been shown to be sensitive to both low oxygen (Föll et al., 1999) and cold shock (2°C) (Robinson and Powell, 2016); additionally, these worms are capable of acclimatizing to the cold to become far more resistant to cold shock (Murray et al., 2007). Toxicity of excess oxygen, however, has not been previously demonstrated in *C. elegans*, as they are capable of surviving and reproducing for up to 50 generations in 100% oxygen at atmospheric pressure (Voorhies and Ward, 2000). Here, we use changes in temperature and oxygen concentration to demonstrate the contribution of oxygen toxicity to cold shock lethality. We also introduce a novel method of inducing oxygen toxicity at room temperature using high pressure oxygen. Finally, we show that manipulation of the cold acclimation pathway induces resistance to room temperature oxygen toxicity, compounds capable of limiting oxidative damage provide resistance to both oxygen toxicity and cold shock, and antioxidant proteins are essential for wild type cold shock survival.

## 2 Materials and methods

### 2.1 Plates and bacteria

Due to the uncontrollable variation in composition of peptone and agar, peptone-free agarose-based plates were used. For 1 L of this solution, 3 g of NaCl and 15 g of agarose was added to 975 mL of agarose and autoclaved for 1 h. After cooling, 1 mL of cholesterol (5 mg/mL in ethanol), 1 mL of 1 M CaCl_2_, 1 mL of 1 M MgSO_4_, 1 mL of uracil (2 mg/mL in water), and 25 mL of 1 M (pH 6.0) KPO_4_ buffer were added. 35 mm and 60 mm plates were filled with 5 mL and 15 mL of solution, respectively, and stored at 4°C. 35 mm plates with additional additives were created by adding concentrated solutions of the additive to the molten agarose. It was necessary to adjust the ascorbate solution to a neutral pH to stay within the KPO_4_ buffering capacity.

*E. coli* (OP50) was grown in 1 L of standard LB medium overnight at 37°C on a shaker running at 200 rpm. The bacteria were collected by centrifugation at 4,000 rpm for 15 min, before resuspension in 40 mL of autoclaved water. This solution was added to 60 mm plates as necessary to maintain worm populations. For 35 mm plates, 100 µL of a 10x dilution of this solution was added to the center of each plate.

### 2.2 Nematode maintenance

Bristol N2, TJ1052 (*age-1*), CB1265 (*unc-104*), TU38 (*deg-1*), BQ1 (*akt-1*), VC289 (*prdx-2*), VC1151 (*prdx-3*), VC754 (*ctl-2*), and RB1633 (*ctl-3*) strains were sourced from the *Caenorhabditis* Genetics Center. Nematodes were grown on 60 mm plates from eggs to adulthood. To produce a new synchronized generation, adults were minced using a razor blade and the released eggs were distributed onto a new plate. Room temperature raised-worms were grown on the bench for 3 days to reach adulthood, while 12°C-raised worms were grown in an incubator for 9 days to reach adulthood. For survival assays, these populations were then washed off the 60 mm plates using water. The adults were then settled and resuspended in water 3 times to remove residual bacteria and juvenile nematodes. From this solution, adults were transferred to 35 mm plates, which had any dead or juvenile nematodes removed from them after drying. When working with additives, nematodes were instead transferred to the control and additive plates as L1s, with dead and juvenile nematodes being removed once the populations had reached adulthood. CB1265 (unc-104) adults were transferred to plates using a platinum wire, as their uncoordinated phenotype prevented them from swimming; this made separating adults from juveniles using the described settling method inefficient, as they settled at similar rates.

### 2.3 Cold shock assays

35 mm nematode plates were placed in sealed containers (Anaeropack Rectangular Jars, Mitsubishi Gas, Japan) modified to have two openings, allowing gas (either room air, 100% nitrogen, or nitrogen with 1,000 or 5,000 ppm oxygen) to be run through each one at 200 cc/min using a mass flow controller (SmartTrak Mass Flow Controller, Sierra Inst., Monterey, CA). Gases were humidified with a bubble chamber prior to reaching the nematodes. These sealed containers were placed into a 2°C incubator. Nematode plates were moved to the benchtop after a time course was completed. Survival was assessed the day after removal from the condition. Nematodes in the nitrogen-room temperature condition were not placed into an incubator; the remainder of the procedure was identical. Instead of using the continuous-flow containers, nematodes in cold HBO were pressurized in a pressure chamber (see “Hyperbaric Oxygen Assays” section of Methods), which was then placed in a 2°C incubator.

### 2.4 Hyperbaric oxygen assays

Nematode plates were placed into a pressure chamber (Stainless Steel 5 L Pressure Vessel, Alloy Products, Waukesha, WI). After sealing, the chamber was flushed with 100% oxygen or 99.5:0.5 N_2_:O_2_ for 90 s by holding the pressure release valve open. The pressure release valve was then closed, and the chambers were pressurized. Chambers were pressurized to 687 kPa in hyperbaric oxygen assays unless otherwise specified. Due to the slight expansion of the pressure chambers caused by the pressurized gas, it was necessary to add additional gas 1–3 min after initial pressurization to restore the desired pressure; pressure was consistent after this. When a time course was completed, the chamber was depressurized to return the animals to room pressure/room air.

### 2.5 Statistical analysis

**FIGURE 1:**
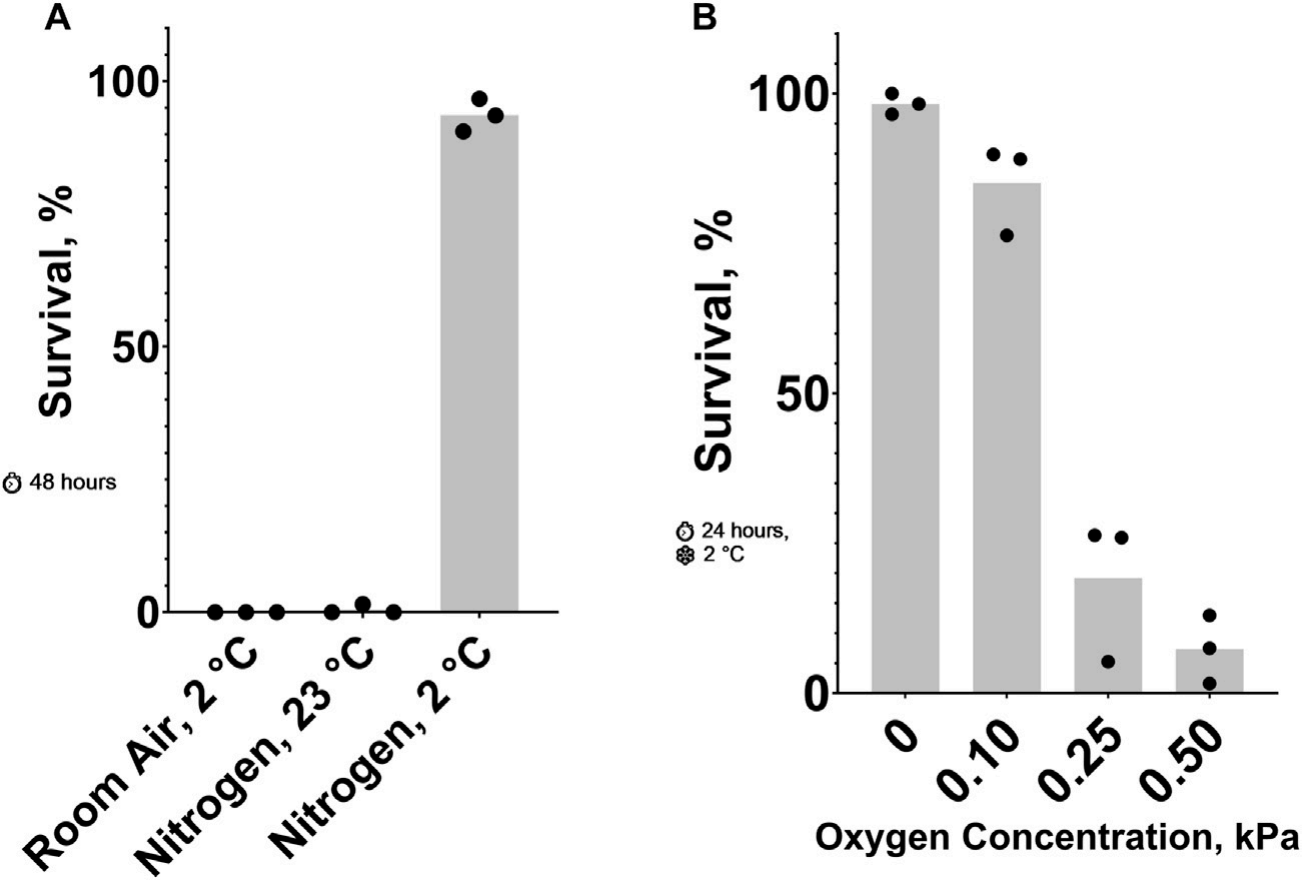
Effect of Oxygen Concentration on ***C.*** *elegans* Cold Shock Survival. **(A)** Survival after 48 h 23°C nitrogen, 2°C room air, and 2°C nitrogen. The probability of no interaction between nitrogen and 2°C is less than 0.05. See ST1 and ST2. **(B)** Survival after a 24-h 2°C cold shock in nitrogen containing low oxygen concentrations. See ST3.

**FIGURE 2:**
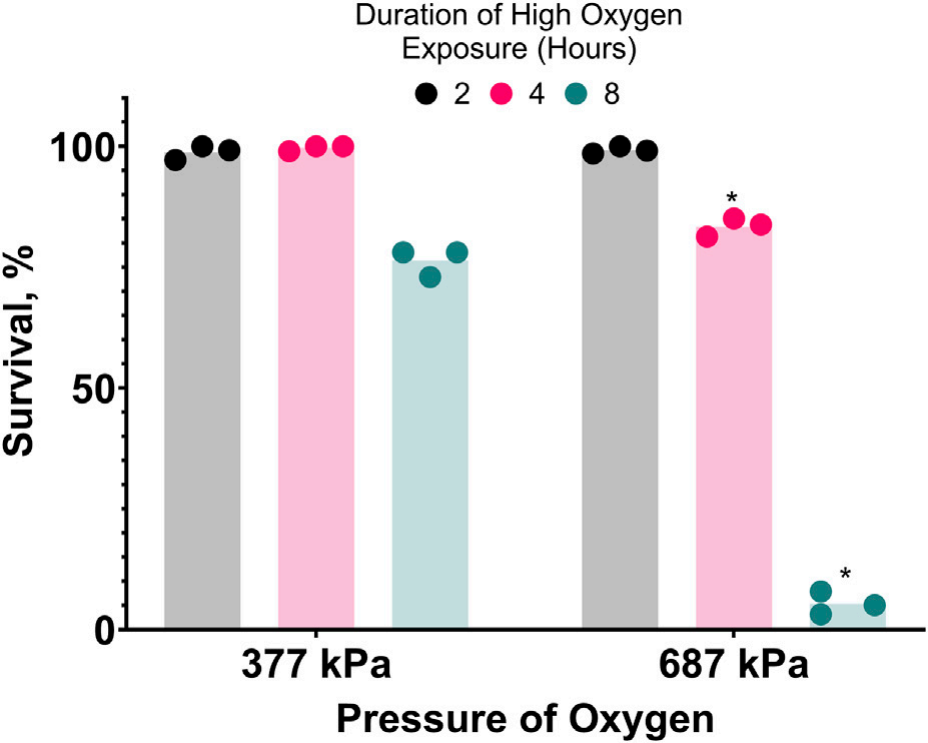
Hyperbaric oxygen (HBO) is toxic. Survival of *C. elegans* in pure oxygen at high pressure. Duration of high oxygen exposure in hours is 2 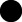, 4 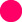, 8 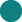. *P < 0.05 when comparing to 377 kPa. See ST4.

**FIGURE 3:**
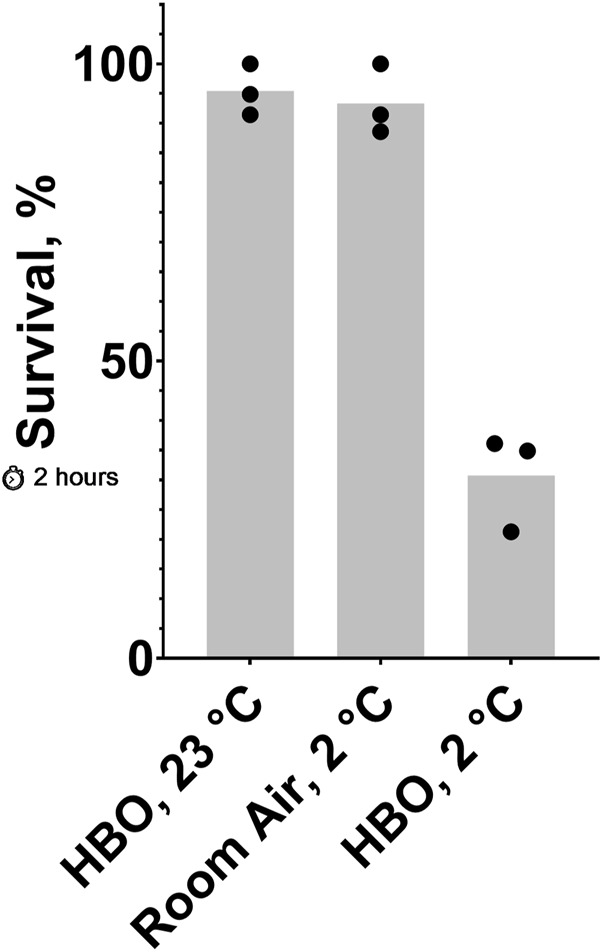
Effect of 2-hour exposure to combined cold shock and HBO. Survival of *C. elegans* after a 2-hour exposure to 23°C HBO, 2°C HBO, or 2°C room air. The probability of no interaction between HBO and 2°C is less than 0.05. See ST5 and ST6.

**FIGURE 4:**
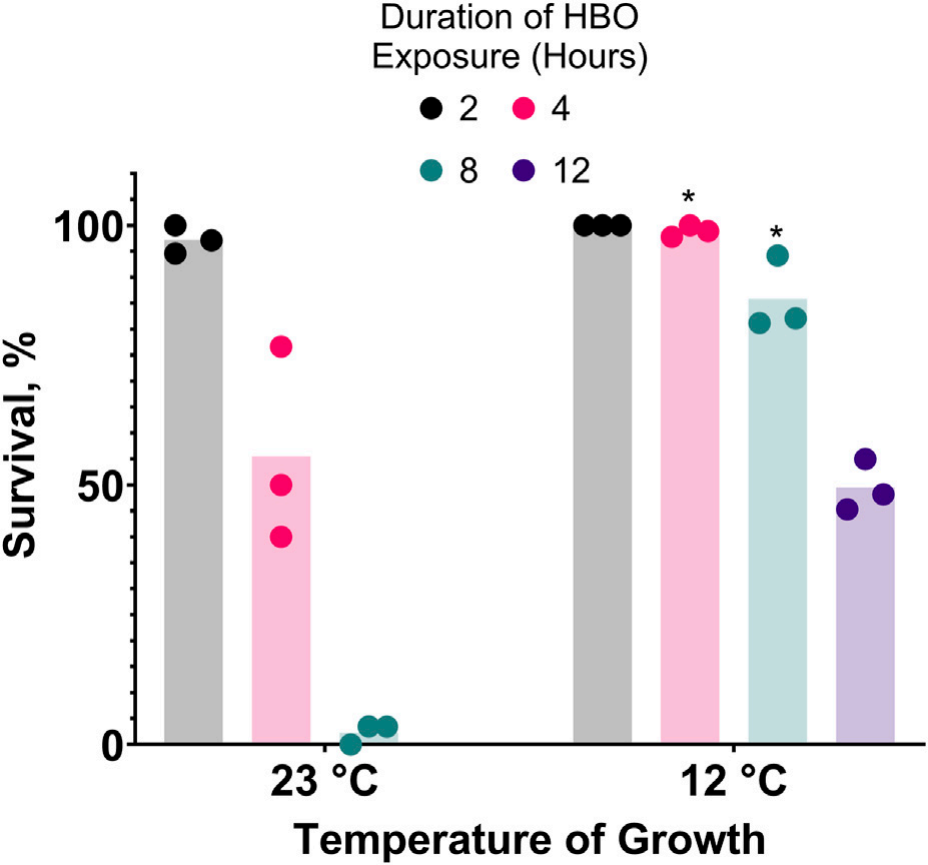
Cold acclimatization provides resistance to hyperbaric oxygen. Survival of animals grown from L1 stage at 23°C or 12°C and subsequently exposed to HBO as adults. Duration of HBO exposure in hours is 2 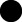, 4 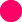, 8 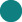, 12 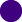. *P < 0.05 when comparing to animals grown at 23°C. See ST7.

**FIGURE 5:**
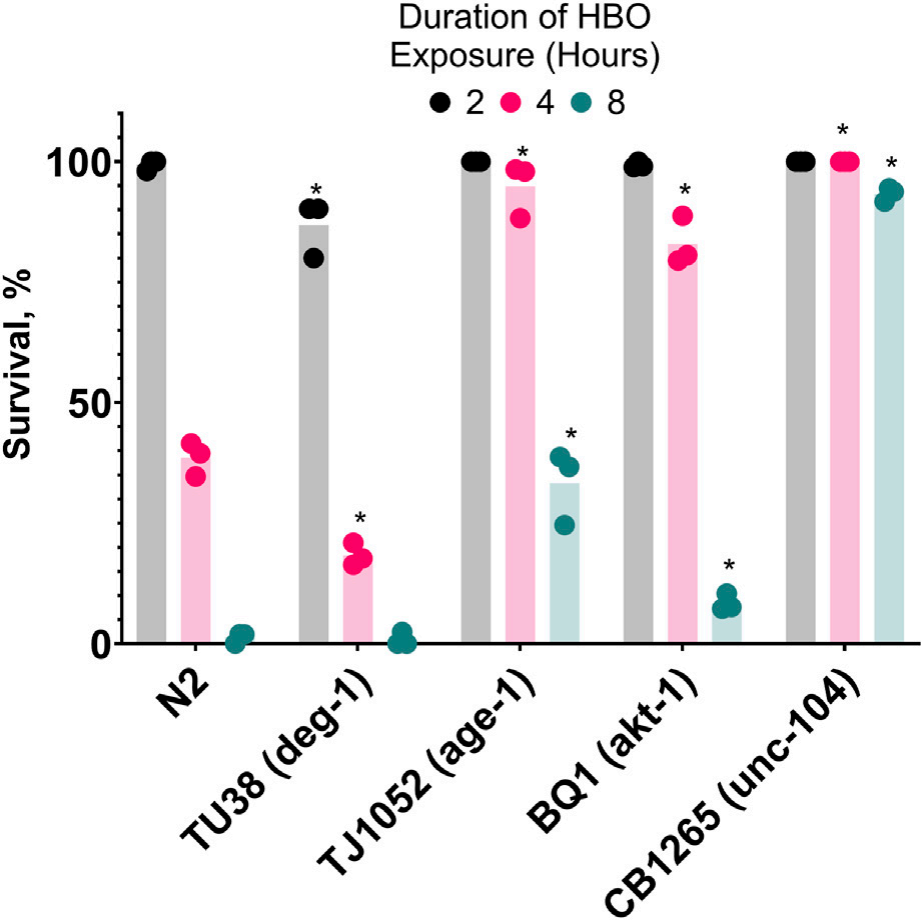
Genetic background affects survival in hyperbaric oxygen. Survival of mutant strains in HBO. Duration of HBO exposure in hours is 2 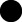, 4 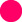, 8 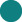. *P < 0.05 when comparing to wild type (N2). See ST8.

**FIGURE 6:**
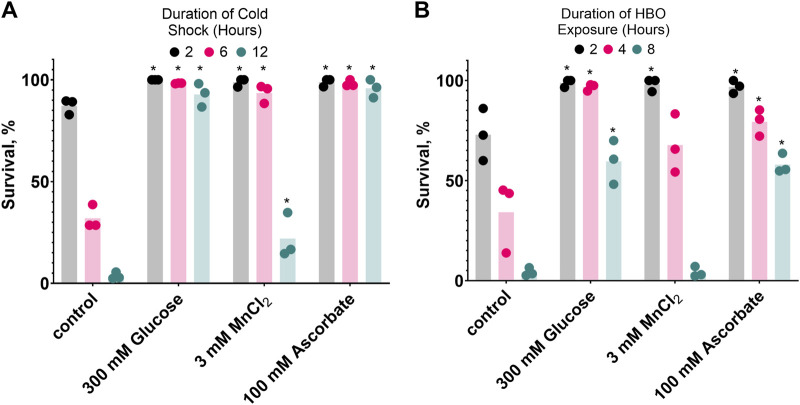
Glucose, MnCl_2_, and ascorbate increase both cold shock and hyperbaric oxygen resistance. Nematodes were grown on plates containing 3 mM MnCl_2_, 300 mM Glucose, or 100 mM Ascorbate. **(A)** Survival in 2°C cold shock, duration in hours 2 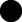, 6 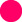, 12 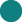. See ST9. **(B)** Survival in HBO, duration in hours 2 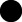, 4 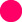, 8 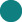. *P < 0.05 when comparing to control (untreated) animals. See ST10.

All assays were performed with three populations of adult *C. elegans* in each condition. All data points in a single figure were collected on the same day. In our supplementary materials, the number alive, number dead, number total, and percent alive are listed for each datapoint, and the P-value, mean percent alive, and standard deviation are listed for each condition. In time courses (Figures 2, 4, 5, 6A, B), the statistical significance of the effect of treatment/genetic background on survival rate was evaluated by using homoscedastic, two-tailed t-tests to compare the survival rates of treated/mutant populations to those of control/wild type populations at the same cold shock/HBO exposure time. In assays showing the effect of combining environmental conditions on survival rate (Figures 1A, 3), a linear regression with 11 degrees of freedom was used to show that the two conditions interacted with each other.

## 3 Results

### 3.1 Oxygen levels that are toxic in the cold

Previously, it has been shown that cold (2°C cold shock) (Robinson and Powell, 2016) and anoxic (100% nitrogen) conditions (Föll et al., 1999) can be lethal to adult *C. elegans*. Our data shows that these conditions kill nearly 100% of animals in 24 and 48 h, respectively. Here, we show that combining these two conditions results in a higher survival rate than either one alone. Figure 1A shows that most nematodes in 23°C nitrogen or 2°C room air died after 48 h, while those in 2°C nitrogen for the same amount of time had an average survival rate of 93%. To further explore this phenomenon, we titrated the oxygen concentration in a 24-hour, 2°C cold shock (Figure 1B). Small changes in oxygen concentration resulted in dramatic changes in survival. More than 85% of nematodes exposed to 0.10 kPa or less of oxygen survived on average; increasing the oxygen concentration to just 0.25 kPa decreased the survival rate to less than 20%.

### 3.2 High oxygen is toxic at room temperature

To further investigate the oxygen-dependent death occurring during cold shock, we attempted to reproduce oxygen toxicity at room temperature. *C. elegans* can complete their life cycle in 100% oxygen at room pressure (Van Voorhies and Ward, 2000). We found that slightly increasing the pressure of pure oxygen to 136 kPa does not kill egg-laying adults, but it does prevent their progeny from developing to adults. Further increasing the pressure of pure oxygen to 377 kPa or 687 kPa (687 kPa of oxygen is hereafter referred to as hyperbaric oxygen/HBO) causes mortality in the first 8 h of exposure (Figure 2). Increasing oxygen pressure was associated with a decrease in survival at all time-points after the first 2 hours. Additionally, the survival rate of nematodes in 687 kPa of pure oxygen negatively correlated with length of exposure at all time-points. Importantly, the mortality observed in these assays was dependent on the partial pressure of oxygen, not total pressure: when a population of 20 egg-laying adults was exposed to a mixture of 99.5% nitrogen and 0.5% oxygen gas raised to 1,103 kPa, a pressure 60% higher than the one causing strong lethality in pure oxygen and containing a partial oxygen pressure of 5.5 kPa, no mortality was observed during their egg laying period and their progeny were able to grow to adulthood under pressure. These results show that oxygen at high concentration is a dose and time-dependent toxin in *C. elegans*.

### 3.3 Low temperature increases the toxicity of hyperbaric oxygen

Because oxygen toxicity appears to be increased in the cold, we predicted that less-lethal exposures to HBO and cold shock could combine to produce high lethality. Here, we show that HBO and cold shock synergize to cause greater lethality than either condition alone (Figure 3). Nematodes exposed to room temperature HBO or cold room air for 2 hours had average survival rates of 95% and 93%, respectively, whereas nematodes exposed to cold HBO for the same amount of time had an average survival rate of just 31%, an almost three-fold decrease from the expected survival rate of 88% if these conditions acted independently of one another.

### 3.4 Cold acclimatization confers resistance to hyperbaric oxygen

Prior acclimatization of *C. elegans* to moderately low temperatures improves their survival during cold shock (Murray et al., 2007). If oxygen toxicity increases in the cold, one would expect that cold acclimatization would increase resistance to oxygen toxicity. We predicted that this resistance could also improve resistance to oxygen toxicity at room temperature HBO. Consistent with this prediction, we show that prior acclimatization of nematodes by growing them at 12°C dramatically increases their capacity for survival in room temperature HBO compared to those grown at room temperature (23°C), effectively tripling the LD_50_ from 4 h to 12 h (Figure 4).

### 3.5 Genes responsible for regulating cold acclimatization affect survival in hyperbaric oxygen

After showing that cold acclimatization increased resistance to HBO, we speculated that manipulating genes related to the activation of cold acclimatization would produce similar effects. It has been previously shown that *age-1* (−), *unc-104* (−), and *akt-1* (−) strains of *C. elegans* have enhanced resistance to cold shock compared to wild type due to constitutive activation of the cold acclimatization pathway (Ohta et al., 2014), while *deg-1* (−) strains are cold acclimatization defective due to this gene’s essential role as a thermosensor (Takagaki et al., 2020). Here, we tested if these mutants also have atypical survival in HBO at room temperature (Figure 5). We found that TJ1052 (*age-1*), BQ1 (*akt-1*), and CB1265 (unc-104), strains exhibited enhanced survival rates in HBO relative to wild type, while the strain TU38 (*deg-1*) had a decreased survival rate, suggesting an overlap between cold tolerance and oxygen tolerance pathways.

### 3.6 Diverse compounds confer resistance to both cold shock and hyperbaric oxygen

If oxidative damage contributes to *C. elegans* mortality during HBO exposure and cold shock, one would expect that compounds capable of reducing oxidative potential could have beneficial effects on survival. We chose to test glucose, which produces a reducing environment as it is metabolized (Fan et al., 2020), manganese (II) chloride, a metal ion that catalyzes the disproportionation of hydrogen peroxide (Stadtman et al., 1990), and ascorbate, an essential antioxidant vitamin (Kawashima et al., 2015). Here, we demonstrate that all three of these compounds enhance the survival of *C. elegans* in both cold shock and HBO (Figure 6). Nematodes were grown from the L1 stage to adulthood on plates containing either 300 mM glucose, 3 mM MnCl_2_, 100 mM ascorbate, or no additive, before being exposed to either HBO or cold shock. All three additives induced increased survival at most timepoints in both conditions. These results further suggest that cold tolerance and oxygen tolerance are overlapping pathways.

### 3.7 Antioxidant proteins are required for wild type cold shock and hyperbaric oxygen survival

**FIGURE 7:**
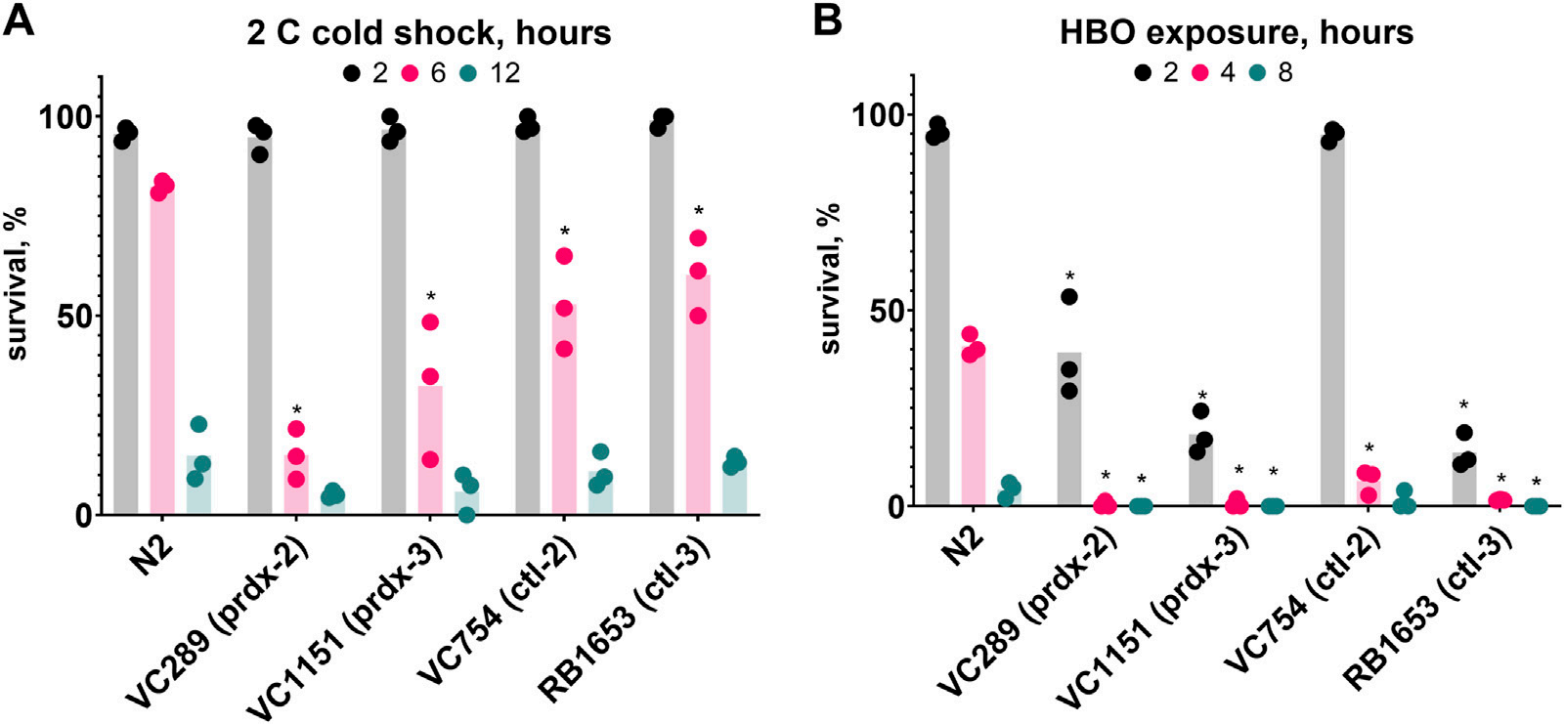
Antioxidant proteins are required for wild type survival during cold shock and HBO exposure. Survival of peroxiredoxin (*prdx*) and catalase (*ctl*) knockout strains relative to wild type. **(A)** Survival in 2°C cold shock, duration in hours 2 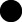, 6 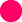, 12 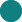. See ST11. **(B)** Survival in HBO, duration in hours 2 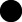, 4 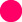, 8 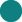. *P < 0.05 when comparing to control (untreated) animals. See ST12.

To further our understanding of the role of oxidative damage in HBO and cold shock death, we investigated the importance of antioxidant proteins in these conditions. To do this, we tested the survival of strains lacking peroxiredoxins and catalases, two classes of protein responsible for the disproportionation of hydrogen peroxide. We found that the strains VC289 (*prdx-2*), VC1151 (*prdx-3*), VC754 (*ctl-2*), and RB1653 (*ctl-3*) all had reduced survival rates relative to wild type during both HBO exposure and cold shock (Figure 7). These results demonstrate the importance of antioxidant proteins for protection from oxygen toxicity and cold shock.

## 4 Discussion

In our work, we showed significant overlap between the pathologies of cold shock and oxygen toxicity in *C. elegans*. We showed that nematodes survived cold anoxia better than room temperature anoxia or cold room air. We demonstrated that HBO could kill nematodes, and that low temperature increased the toxicity of HBO. We also showed that we could improve HBO survival by cold-acclimatizing nematodes, and that mutations to genes in the cold acclimatization pathway affected HBO tolerance. Finally, we identified naturally occurring and physiologically relevant compounds that improved survival limits in both cold shock and HBO, as well as demonstrating that antioxidant proteins were necessary for wild-type survival in these conditions. These results suggest that oxygen toxicity is increased in the cold and contributes to cold-related death in *C. elegans*.

The increased toxicity of oxygen in the cold is likely the result of a variety of factors. Decreasing the temperature from 23°C to 2°C increases the solubility of oxygen in water by approximately 57%. This appears insufficient to induce oxygen toxicity on its own since a more than 5-fold increase above atmospheric oxygen concentrations did not increase lethality, and only slowed growth. However, other processes may account for the toxicity we observed. The rate-reduction of temperature-sensitive enzymes responsible for the neutralization of reactive oxygen species (ROS) and control of redox potential may limit the ability to prevent oxidative damage. Notably, *C. elegans* decrease oxygen consumption as temperature decreases (Van Voorhies and Ward, 1999), which may be an adaptation to reduce the potential for oxidative damage; oxidative metabolism has been implicated as a way that organisms produce ROS from oxygen (Korge et al., 2016). Additionally, if oxygen-dependent processes proceed while temperature-sensitive ones are slowed or stopped, the mismatch between these processes could lead to fatal miscoordination. For example, our lab has previously shown that cold-shocked 2-cell *C. elegans* embryos were incapable of cytokinesis in the cold but would continue to produce up to 8 centrioles in each blastomere, resulting in failure of chromosome segregation, aneuploidy, and death upon restoration of the cell cycle when returned to room temperature (Chan et al., 2010). Embryos that were anoxic during the cold exposure did not produce excess centrioles and, as a result, were viable upon restoration of room air and temperature. The dramatic difference in cold shock survival rates between nematodes exposed to 0.1 and 0.25 kPa oxygen is further indication that limiting oxidative activity is beneficial in the cold; it has been previously shown that the metabolic rate of *C. elegans* increases sharply between oxygen concentrations of 0 and 0.5 kPa (Van Voorhies and Ward, 2000). Anoxia may limit oxidative activity that is toxic in the cold, while the cold may slow the rate at which sugars necessary for anaerobic metabolism are consumed, resulting in the observed synergy between these conditions.

The resistance to HBO shown by *C. elegans* acclimatized to cold suggests that oxygen toxicity in the cold is significant enough to provide evolutionary pressure for the development of mechanisms within the cold acclimatization pathway to remain alive in the cold. In our research, we focused on genes related to cold acclimatization regulation to emphasize the relationship between activation of this pathway and oxygen toxicity resistance; future research could focus on the specific genes affected by this pathway that confer this resistance. ASJ neurons use insulin to prevent other neurons from initiating a cold-tolerance response; as the temperature drops, the ASJ neurons produce less insulin (Ohta et al., 2014). ASJ neurons are incapable of transmitting this signal in worms without *unc-104* (a kinesin), while *age-1* (−) and *akt-1* (−) worms have neurons that are insensitive to the insulin signal. Notably, *age-1* (−) nematodes have a lower metabolic rate than wild type (Van Voorhies and Ward, 1999), a phenotype which may confer resistance to both cold shock and HBO. The *deg-1* gene product is a sodium channel that acts as a thermoreceptor in ASG neurons, which are responsible for positive regulation of cold tolerance (Takagaki et al., 2020). Our results show that this gene is also necessary for stress response to high oxygen. Scnn1a, the rat ortholog of *deg-1* (Bult and Sternberg, 2023), shows increased gene expression and protein activity in the alveolar cells of hyperoxic rats (Yue et al., 1995). If *deg-1* activity is similarly affected by high oxygen, it could explain this gene’s importance to HBO tolerance. Further research is necessary to determine if oxygen concentration affects *deg-1*.

The finding that glucose, manganese (II), and ascorbate can protect nematodes from HBO and cold shock provides evidence that diverse strategies for managing oxidative damage protect *C. elegans* in both of these conditions. Ascorbate and manganese (II) have both been implicated in the neutralization of ROS, a potential source of HBO and cold shock toxicity. Ascorbate acts as a direct antioxidant against ROS (Kawashima et al., 2015), as well as acting as a cofactor in a variety of enzymes (Doseděl et al., 2021) necessary for ROS-reducing activities, such as carnitine production (Lee et al., 2022). Manganese (II) is used by many *Lactobacillus* strains in place of superoxide dismutase (Archibald and Fridovich, 1981) and acts as a catalyst for hydrogen peroxide dismutation in the presence of biological levels of bicarbonate (Stadtman et al., 1990). Glucose may protect nematodes from cold shock and HBO through a variety of mechanisms. Glucose supplementation of *C. elegans* has been shown to increase the activity of proteins responsible for coordinating the unfolded protein response such as IRE-1 (Beaudoin-Chabot et al., 2022), a protein kinase found in the endoplasmic reticulum of neurons that is necessary for wild-type cold shock survival (Dudkevich et al., 2022). Further research is necessary to determine the role of IRE-1 and the unfolded protein response in HBO survival. Glucose supplementation may also affect the intracellular oxidation state; in human cells, it is known that hyperglycemia (Fan et al., 2020) and anoxia (Garafalo et al., 1988) increase the NADH/NAD + ratio, while hypothermia decreases it (Eskla et al., 2022). The highly oxidizing environment of HBO likely reduces this ratio as well. The reductive pressure produced by glucose supplementation may counteract the oxidative environments produced by HBO and cold shock, resulting in the enhanced tolerance to these environments that we observed. Likewise, our own research showing that combining two oxidative conditions (HBO and cold shock, Figure 3) resulted in decreased survival is in line with previous studies demonstrating lower survival in two reductive conditions (hyperglycemia and anoxia, Garcia et al., 2015). Future research is necessary to confirm the effects of these environments on the intracellular oxidation state.

The finding that four different peroxiredoxin and catalase knockout strains all have reduced survival in the cold relative to wild type further suggests the importance of oxidative stress response in this environment. Peroxiredoxin is oxidized by H_2_O_2_, producing H_2_O as a byproduct; it is later reduced by thioredoxin. Catalase catalyzes the reaction of 2 H_2_O_2_ to produce 2 H_2_O and O_2_; it can also catalyze the oxidation of toxins by H_2_O_2_. *Prdx-2* localizes to the cytoplasm (Xia et al., 2023), *ctl-2* to the peroxisomes (Petriv and Ruchabinski, 2004), and *prdx-3* to the mitochondria (Ranjan et al., 2013); the localization of *ctl-3* has not been experimentally determined to our knowledge. *Ctl-3* possesses an intrinsically disordered region (Doğan et al., 2016) that may increase its affinity for unfolded proteins, suggesting that it may be involved in preventing oxidation of unfolded proteins. The importance of peroxiredoxin to cold tolerance has not been previously demonstrated; catalase, however, has been used to limit the damage caused by cold-induced ischemia in the legs of live rabbits (Das et al., 1991). Determining the role each of these proteins have in reducing cold-induced oxidative stress will require further research.

Is oxygen toxicity in the cold a nematode-specific phenomenon, or is it found in other animals? Animals ranging from protozoans to mammals are known to decrease their body temperature in hypoxic conditions (Wood, 1991). While not directly providing evidence of oxygen toxicity, this adaptation mirrors our result regarding survival of nematodes in cold nitrogen. In humans, recent analysis has shown that hypothermia patients with hyperoxic arterial oxygen concentrations had a 28-day mortality rate twice that of normoxic patients (Yamamoto et al., 2023). Further research to understand if the observations made here extend to other animals and humans may improve our ability to use oxygen concentration and temperature to improve clinical outcomes.

## Data Availability

The original contributions presented in the study are included in the article/Supplementary Material, further inquiries can be directed to the corresponding author.
