# Supplementary material for: Oxygen is toxic in the cold in C. elegans
Source: Front Physiol. 2024 Dec 24;15:1471249. doi: 10.3389/fphys.2024.1471249 (PMC11703811; doi:10.3389/fphys.2024.1471249)
Supplement: Supplementary file 1 [file DataSheet1.docx]

Supplementary Material

# Supplementary Materials for Figure 1A

## Supplementary Table 1 – Raw Data for Figure 1A

| Exposure Time | 48 hours | | | | | | | | | | | | | |
| --- | --- | --- | --- | --- | --- | --- | --- | --- | --- | --- | --- | --- | --- | --- |
|  | Replicate 1 | | | | Replicate 2 | | | | Replicate 3 | | | | Summary | |
| Condition | total | # Alive | # Dead | % Alive | total | # Alive | # Dead | % Alive | total | # Alive | # Dead | % Alive | Mean | Std. dev. |
| Room Air, 2 °C | 66 | 0 | 66 | 0.00% | 61 | 0 | 61 | 0.00% | 54 | 0 | 54 | 0.00% | 0.00% | 0.00% |
| Nitrogen, 23 °C | 64 | 0 | 64 | 0.00% | 70 | 0 | 70 | 0.00% | 66 | 1 | 65 | 1.52% | 0.50% | 0.71% |
| Nitrogen, 2 °C | 62 | 58 | 4 | 93.55% | 74 | 67 | 7 | 90.54% | 121 | 117 | 4 | 96.69% | 93.59% | 2.51% |

## Supplementary Table 2 – Regression for Interaction of Nitrogen and 2 °C in Figure 1A

| Condition | Replicate | Nitrogen | 2 °C | Nitrogen + 2 °C | Percent Survival |
| --- | --- | --- | --- | --- | --- |
| Nitrogen, 2 °C | 1 | 1 | 1 | 1 | 93.55% |
|  | 2 | 1 | 1 | 1 | 90.54% |
|  | 3 | 1 | 1 | 1 | 96.69% |
| Nitrogen, 23 °C | 1 | 1 | 0 | 0 | 0.00% |
|  | 2 | 1 | 0 | 0 | 0.00% |
|  | 3 | 1 | 0 | 0 | 1.52% |
| Room Air, 2 °C | 1 | 0 | 1 | 0 | 0.00% |
|  | 2 | 0 | 1 | 0 | 0.00% |
|  | 3 | 0 | 1 | 0 | 0.00% |
| Room Air, 23 °C | 1 | 0 | 0 | 0 | 100.00% |
|  | 2 | 0 | 0 | 0 | 100.00% |
|  | 3 | 0 | 0 | 0 | 100.00% |

Supplementary table 2, cont.

| Regression Statistics | |  |  |  |  |  |  |  |
| --- | --- | --- | --- | --- | --- | --- | --- | --- |
| Multiple R | 0.999635 |  |  |  |  |  |  |  |
| R Square | 0.99927 |  |  |  |  |  |  |  |
| Adjusted R Square | 0.998997 |  |  |  |  |  |  |  |
| Standard Error | 0.015995 |  |  |  |  |  |  |  |
| Observations | 12 |  |  |  |  |  |  |  |
| ANOVA |  |  |  |  |  |  |  |  |
|  | df | SS | MS | F | Significance F |  |  |  |
| Regression | 3 | 2.802455 | 0.934152 | 3651.261 | 6.98E-13 |  |  |  |
| Residual | 8 | 0.002047 | 0.000256 |  |  |  |  |  |
| Total | 11 | 2.804502 |  |  |  |  |  |  |
|  | Coefficients | Standard Error | t Stat | P-value | Lower 95% | Upper 95% | Lower 95.0% | Upper 95.0% |
| Intercept | 1 | 0.009235 | 108.2863 | 5.91E-14 | 0.978705 | 1.021295 | 0.978705 | 1.021295 |
| Nitrogen | -0.99495 | 0.01306 | -76.1832 | 9.82E-13 | -1.02507 | -0.96483 | -1.02507 | -0.96483 |
| 2 °C | -1 | 0.01306 | -76.57 | 9.43E-13 | -1.03012 | -0.96988 | -1.03012 | -0.96988 |
| Nitrogen + 2 °C | 1.930893 | 0.01847 | 104.5446 | 7.83E-14 | 1.888302 | 1.973484 | 1.888302 | 1.973484 |

The probability of no interaction between the nitrogen and 2 °C conditions based on our data is 7.83x10^-14^.

# Supplementary Table 3 – Raw Data for Figure 1B

| Exposure Time and Temp | 24 hours, 2 °C | | | | | | | | | | | | | |
| --- | --- | --- | --- | --- | --- | --- | --- | --- | --- | --- | --- | --- | --- | --- |
|  | Replicate 1 | | | | Replicate 2 | | | | Replicate 3 | | | | Summary | |
| Oxygen Conc, kPa | total | # Alive | # Dead | Mean | Mean | # Alive | # Dead | % Alive | total | # Alive | # Dead | % Alive | Mean | Std. dev. |
| 0 | 48 | 48 | 0 | 100.00% | 58 | 57 | 1 | 98.28% | 58 | 56 | 2 | 96.55% | 98.28% | 1.41% |
| 0.1 | 38 | 29 | 9 | 76.32% | 73 | 65 | 8 | 89.04% | 59 | 53 | 6 | 89.83% | 85.07% | 6.19% |
| 0.25 | 38 | 2 | 36 | 5.26% | 54 | 14 | 40 | 25.93% | 57 | 15 | 42 | 26.32% | 19.17% | 9.83% |
| 0.5 | 77 | 10 | 67 | 12.99% | 40 | 3 | 37 | 7.50% | 62 | 1 | 61 | 1.61% | 7.37% | 4.64% |

# Supplementary Table 4 – Data and Analysis for Figure 2

| Exposure Time | 2 hours | | | | | | | | | | | | | |
| --- | --- | --- | --- | --- | --- | --- | --- | --- | --- | --- | --- | --- | --- | --- |
|  | Replicate 1 | | | | Replicate 2 | | | | Replicate 3 | | | | Summary | |
| Oxygen Pressure, kPa | total | # Alive | # Dead | % Alive | total | # Alive | # Dead | % Alive | total | # Alive | # Dead | % Alive | Mean | Std. dev. |
| 687 | 73 | 73 | 0 | 100.00% | 118 | 117 | 1 | 99.15% | 68 | 67 | 1 | 98.53% | 99.23% | 0.60% |
| 377 | 115 | 115 | 0 | 100.00% | 131 | 130 | 1 | 99.24% | 107 | 104 | 3 | 97.20% | 98.81% | 1.18% |

Supplementary table 4, cont.

| Exposure Time | 4 hours | | | | | | | | | | | | | |
| --- | --- | --- | --- | --- | --- | --- | --- | --- | --- | --- | --- | --- | --- | --- |
|  | Replicate 1 | | | | Replicate 2 | | | | Replicate 3 | | | | Summary | |
| Oxygen Pressure, kPa | total | # Alive | # Dead | % Alive | total | # Alive | # Dead | % Alive | total | # Alive | # Dead | % Alive | Mean | Std. dev. |
| 687 | 99 | 83 | 16 | 83.84% | 91 | 74 | 17 | 81.32% | 67 | 57 | 10 | 85.07% | 83.41% | 1.56% |
| 377 | 109 | 109 | 0 | 100.00% | 100 | 99 | 1 | 99.00% | 129 | 129 | 0 | 100.00% | 99.67% | 0.47% |

Supplementary table 4, cont.

| Exposure Time | 8 hours | | | | | | | | | | | | | |
| --- | --- | --- | --- | --- | --- | --- | --- | --- | --- | --- | --- | --- | --- | --- |
|  | Replicate 1 | | | | Replicate 2 | | | | Replicate 3 | | | | Summary | |
| Oxygen Pressure, kPa | total | # Alive | # Dead | % Alive | total | # Alive | # Dead | % Alive | total | # Alive | # Dead | % Alive | Mean | Std. dev. |
| 687 | 126 | 4 | 122 | 3.17% | 158 | 8 | 150 | 5.06% | 177 | 14 | 163 | 7.91% | 5.38% | 1.95% |
| 377 | 148 | 108 | 40 | 72.97% | 73 | 57 | 16 | 78.08% | 114 | 89 | 25 | 78.07% | 76.38% | 2.41% |

Supplementary table 4, cont.

| T-test relative to 377 kPa condition | 2 hours | 4 hours | 8 hours |
| --- | --- | --- | --- |
| 687 kPa | 0.680450616 | 0.000147549 | 5.37981E-06 |

# Supplementary Materials for Figure 3

## Supplementary Table 5 – Raw Data for Figure 3

| Exposure Time | 2 hours | | | | | | | | | | | | | |
| --- | --- | --- | --- | --- | --- | --- | --- | --- | --- | --- | --- | --- | --- | --- |
|  | Replicate 1 | | | | Replicate 2 | | | | Replicate 3 | | | | Summary | |
| Condition | total | # Alive | # Dead | % Alive | total | # Alive | # Dead | % Alive | total | # Alive | # Dead | % Alive | Mean | Std. dev. |
| HBO, 23 °C | 39 | 37 | 2 | 94.87% | 29 | 29 | 0 | 100.00% | 35 | 32 | 3 | 91.43% | 95.43% | 3.52% |
| HBO, 2 °C | 47 | 10 | 37 | 21.28% | 36 | 13 | 23 | 36.11% | 43 | 15 | 28 | 34.88% | 30.76% | 6.72% |
| Room Air, 2 °C | 35 | 32 | 3 | 91.43% | 38 | 38 | 0 | 100.00% | 35 | 31 | 4 | 88.57% | 93.33% | 4.86% |

## Supplementary Table 6 – Regression for Interaction of HBO and 2 °C in Figure 3

| Condition | Replicate | HBO | 2 °C | HBO + 2 °C | Percent Survival |
| --- | --- | --- | --- | --- | --- |
| HBO, 2 °C | 1 | 1 | 1 | 1 | 21.28% |
|  | 2 | 1 | 1 | 1 | 36.11% |
|  | 3 | 1 | 1 | 1 | 34.88% |
| HBO, 23 °C | 1 | 1 | 0 | 0 | 94.87% |
|  | 2 | 1 | 0 | 0 | 100.00% |
|  | 3 | 1 | 0 | 0 | 91.43% |
| Room Air, 2 °C | 1 | 0 | 1 | 0 | 91.43% |
|  | 2 | 0 | 1 | 0 | 100.00% |
|  | 3 | 0 | 1 | 0 | 88.57% |
| Room Air, 23 °C | 1 | 0 | 0 | 0 | 100.00% |
|  | 2 | 0 | 0 | 0 | 100.00% |
|  | 3 | 0 | 0 | 0 | 100.00% |

Supplementary table 6, cont.

| Regression Statistics | |  |  |  |  |  |  |  |
| --- | --- | --- | --- | --- | --- | --- | --- | --- |
| Multiple R | 0.987706171 |  |  |  |  |  |  |  |
| R Square | 0.97556348 |  |  |  |  |  |  |  |
| Adjusted R Square | 0.966399785 |  |  |  |  |  |  |  |
| Standard Error | 0.055173656 |  |  |  |  |  |  |  |
| Observations | 12 |  |  |  |  |  |  |  |
| ANOVA |  |  |  |  |  |  |  |  |
|  | df | SS | MS | F | Significance F |  |  |  |
| Regression | 3 | 0.972231501 | 0.324077 | 106.4596 | 8.689E-07 |  |  |  |
| Residual | 8 | 0.024353058 | 0.003044 |  |  |  |  |  |
| Total | 11 | 0.996584559 |  |  |  |  |  |  |
|  | Coefficients | Standard Error | t Stat | P-value | Lower 95% | Upper 95% | Lower 95.0% | Upper 95.0% |
| Intercept | 1 | 0.031854525 | 31.39271 | 1.15E-09 | 0.926543334 | 1.073456666 | 0.926543334 | 1.073456666 |
| HBO | -0.045665446 | 0.045049101 | -1.01368 | 0.340408 | -0.14954886 | 0.058217968 | -0.14954886 | 0.058217968 |
| 2 °C | -0.066666667 | 0.045049101 | -1.47987 | 0.177176 | -0.170550081 | 0.037216747 | -0.170550081 | 0.037216747 |
| HBO + 2 °C | -0.580096462 | 0.06370905 | -9.1054 | 1.7E-05 | -0.727009795 | -0.433183129 | -0.727009795 | -0.433183129 |

The probability of no interaction between the HBO and 2 °C conditions based on our data is 1.7x10^-5^.

# Supplementary Table 7 – Data and Analysis for Figure 4

| HBO Exposure Time | 2 hours | | | | | | | | | | | | | |
| --- | --- | --- | --- | --- | --- | --- | --- | --- | --- | --- | --- | --- | --- | --- |
|  | Replicate 1 | | | | Replicate 2 | | | | Replicate 3 | | | | Summary | |
| Temperature of Growth | total | # Alive | # Dead | % Alive | total | # Alive | # Dead | % Alive | total | # Alive | # Dead | % Alive | Mean | Std. dev. |
| 23 °C | 35 | 34 | 1 | 97.14% | 93 | 88 | 5 | 94.62% | 47 | 47 | 0 | 100.00% | 97.26% | 2.20% |
| 12 °C | 84 | 84 | 0 | 100.00% | 43 | 43 | 0 | 100.00% | 50 | 50 | 0 | 100.00% | 100.00% | 0.00% |

Supplementary Table 7, cont.

| HBO Exposure Time | 4 hours | | | | | | | | | | | | | |
| --- | --- | --- | --- | --- | --- | --- | --- | --- | --- | --- | --- | --- | --- | --- |
|  | Replicate 1 | | | | Replicate 2 | | | | Replicate 3 | | | | Summary | |
| Temperature of Growth | total | # Alive | # Dead | % Alive | total | # Alive | # Dead | % Alive | total | # Alive | # Dead | % Alive | Mean | Std. dev. |
| 23 °C | 20 | 8 | 12 | 40.00% | 40 | 20 | 20 | 50.00% | 30 | 23 | 7 | 76.67% | 55.56% | 15.48% |
| 12 °C | 87 | 86 | 1 | 98.90% | 52 | 52 | 0 | 100.00% | 46 | 45 | 1 | 97.80% | 98.90% | 0.90% |

Supplementary table 7, cont.

| HBO Exposure Time | 8 hours | | | | | | | | | | | | | |
| --- | --- | --- | --- | --- | --- | --- | --- | --- | --- | --- | --- | --- | --- | --- |
|  | Replicate 1 | | | | Replicate 2 | | | | Replicate 3 | | | | Summary | |
| Temperature of Growth | total | # Alive | # Dead | % Alive | total | # Alive | # Dead | % Alive | total | # Alive | # Dead | % Alive | Mean | Std. dev. |
| 23 °C | 29 | 1 | 28 | 3.45% | 86 | 3 | 83 | 3.49% | 48 | 0 | 48 | 0.00% | 2.31% | 1.64% |
| 12 °C | 78 | 64 | 14 | 82.10% | 69 | 65 | 4 | 94.20% | 69 | 56 | 13 | 81.20% | 85.83% | 5.93% |

Supplementary table 7, cont.

| HBO Exposure Time | 12 hours | | | | | | | | | | | | | |
| --- | --- | --- | --- | --- | --- | --- | --- | --- | --- | --- | --- | --- | --- | --- |
|  | Replicate 1 | | | | Replicate 2 | | | | Replicate 3 | | | | Summary | |
| Temperature of Growth | total | # Alive | # Dead | % Alive | total | # Alive | # Dead | % Alive | total | # Alive | # Dead | % Alive | Mean | Std. dev. |
| 23 °C | n/a | n/a | n/a | n/a | n/a | n/a | n/a | n/a | n/a | n/a | n/a | n/a | n/a | n/a |
| 12 °C | 56 | 27 | 29 | 48.20% | 64 | 29 | 35 | 45.30% | 80 | 44 | 36 | 55.00% | 49.50% | 4.07% |

Supplementary table 7, cont.

| T-test relative to 23 °C growth temperature | 2 hours | 4 hours | 8 hours |
| --- | --- | --- | --- |
| 12 °C | 0.151938877 | 0.016758715 | 4.32803E-05 |

# Supplementary Table 8 – Data and Analysis for Figure 5

| HBO Exposure Time | 2 hours | | | | | | | | | | | | | |
| --- | --- | --- | --- | --- | --- | --- | --- | --- | --- | --- | --- | --- | --- | --- |
|  | Replicate 1 | | | | Replicate 2 | | | | Replicate 3 | | | | Summary | |
| Strain | total | # Alive | # Dead | % Alive | total | # Alive | # Dead | % Alive | total | # Alive | # Dead | % Alive | Mean | Std. dev. |
| N2 | 42 | 42 | 0 | 100.00% | 51 | 50 | 1 | 98.04% | 52 | 52 | 0 | 100.00% | 99.35% | 0.92% |
| TU38 | 51 | 46 | 5 | 90.20% | 40 | 32 | 8 | 80.00% | 61 | 55 | 6 | 90.16% | 86.79% | 4.80% |
| TJ1052 | 54 | 54 | 0 | 100.00% | 53 | 53 | 0 | 100.00% | 54 | 54 | 0 | 100.00% | 100.00% | 0.00% |
| BQ1 | 87 | 86 | 1 | 98.85% | 103 | 102 | 1 | 99.03% | 60 | 60 | 0 | 100.00% | 99.29% | 0.51% |
| CB1265 | 18 | 18 | 0 | 100.00% | 14 | 14 | 0 | 100.00% | 12 | 12 | 0 | 100.00% | 100.00% | 0.00% |

Supplementary Table 8, cont.

| HBO Exposure Time | 4 hours | | | | | | | | | | | | | |
| --- | --- | --- | --- | --- | --- | --- | --- | --- | --- | --- | --- | --- | --- | --- |
|  | Replicate 1 | | | | Replicate 2 | | | | Replicate 3 | | | | Summary | |
| Strain | total | # Alive | # Dead | % Alive | total | # Alive | # Dead | % Alive | total | # Alive | # Dead | % Alive | Mean | Std. dev. |
| N2 | 53 | 22 | 31 | 41.51% | 38 | 15 | 23 | 39.47% | 49 | 17 | 32 | 34.69% | 38.56% | 2.86% |
| TU38 | 61 | 10 | 51 | 16.39% | 79 | 14 | 65 | 17.72% | 43 | 9 | 34 | 20.93% | 18.35% | 1.90% |
| TJ1052 | 34 | 30 | 4 | 88.24% | 60 | 59 | 1 | 98.33% | 48 | 47 | 1 | 97.92% | 94.83% | 4.67% |
| BQ1 | 67 | 54 | 13 | 80.60% | 78 | 62 | 16 | 79.49% | 80 | 71 | 9 | 88.75% | 82.94% | 4.13% |
| CB1265 | 24 | 24 | 0 | 100.00% | 16 | 16 | 0 | 100.00% | 17 | 17 | 0 | 100.00% | 100.00% | 0.00% |

Supplementary Table 8, cont.

| HBO Exposure Time | 8 hours | | | | | | | | | | | | | |
| --- | --- | --- | --- | --- | --- | --- | --- | --- | --- | --- | --- | --- | --- | --- |
|  | Replicate 1 | | | | Replicate 2 | | | | Replicate 3 | | | | Summary | |
| Strain | total | # Alive | # Dead | % Alive | total | # Alive | # Dead | % Alive | total | # Alive | # Dead | % Alive | Mean | Std. dev. |
| N2 | 52 | 1 | 51 | 1.92% | 49 | 0 | 49 | 0.00% | 51 | 1 | 50 | 1.96% | 1.29% | 0.92% |
| TU38 | 58 | 0 | 58 | 0.00% | 41 | 1 | 40 | 2.44% | 71 | 0 | 71 | 0.00% | 0.81% | 1.15% |
| TJ1052 | 65 | 16 | 49 | 24.62% | 62 | 24 | 38 | 38.71% | 60 | 22 | 38 | 36.67% | 33.33% | 6.22% |
| BQ1 | 92 | 7 | 85 | 7.61% | 83 | 6 | 77 | 7.23% | 125 | 13 | 112 | 10.40% | 8.41% | 1.41% |
| CB1265 | 12 | 11 | 1 | 91.67% | 16 | 15 | 1 | 93.75% | 18 | 17 | 1 | 94.44% | 93.29% | 1.18% |

Supplementary table 8, cont.

| T-test relative to N2 | 2 hours | 4 hours | 8 hours |
| --- | --- | --- | --- |
| TU38 | 0.022072 | 0.001137 | 0.6671646 |
| TJ1052 | 0.373901 | 0.00013 | 0.0019643 |
| BQ1 | 0.946514 | 0.000236 | 0.0039398 |
| CB1265 | 0.373901 | 6.96E-06 | 1.042E-07 |

# Supplementary Table 9 – Data and Analysis for Figure 6A

| 2 °C Cold Shock Duration | 2 hours | | | | | | | | | | | | | |
| --- | --- | --- | --- | --- | --- | --- | --- | --- | --- | --- | --- | --- | --- | --- |
|  | Replicate 1 | | | | Replicate 2 | | | | Replicate 3 | | | | Summary | |
| Strain | total | # Alive | # Dead | % Alive | total | # Alive | # Dead | % Alive | total | # Alive | # Dead | % Alive | Mean | Std. dev. |
| Control | 48 | 43 | 5 | 89.58% | 37 | 33 | 4 | 89.19% | 35 | 29 | 6 | 82.86% | 87.21% | 3.08% |
| 300 mM Glucose | 45 | 45 | 0 | 100.00% | 56 | 56 | 0 | 100.00% | 31 | 31 | 0 | 100.00% | 100.00% | 0.00% |
| 3 mM MnCl_2_ | 30 | 30 | 0 | 100.00% | 50 | 50 | 0 | 100.00% | 57 | 55 | 2 | 96.49% | 98.83% | 1.65% |
| 100 mM Ascorbate | 59 | 57 | 2 | 96.61% | 31 | 31 | 0 | 100.00% | 32 | 32 | 0 | 100.00% | 98.87% | 1.60% |

Supplementary Table 9, cont.

| 2 °C Cold Shock Duration | 6 hours | | | | | | | | | | | | | |
| --- | --- | --- | --- | --- | --- | --- | --- | --- | --- | --- | --- | --- | --- | --- |
|  | Replicate 1 | | | | Replicate 2 | | | | Replicate 3 | | | | Summary | |
| Strain | total | # Alive | # Dead | % Alive | total | # Alive | # Dead | % Alive | total | # Alive | # Dead | % Alive | Mean | Std. dev. |
| Control | 31 | 12 | 19 | 38.71% | 49 | 14 | 35 | 28.57% | 56 | 16 | 40 | 28.57% | 31.95% | 4.78% |
| 300 mM Glucose | 61 | 60 | 1 | 98.36% | 63 | 62 | 1 | 98.41% | 52 | 51 | 1 | 98.07% | 98.28% | 0.15% |
| 3 mM MnCl_2_ | 43 | 38 | 5 | 88.37% | 30 | 29 | 1 | 96.67% | 46 | 44 | 2 | 95.65% | 93.56% | 3.69% |
| 100 mM Ascorbate | 37 | 36 | 1 | 97.30% | 36 | 35 | 1 | 97.22% | 45 | 45 | 0 | 100.00% | 98.17% | 1.29% |

Supplementary Table 9, cont.

| 2 °C Cold Shock Duration | 12 hours | | | | | | | | | | | | | |
| --- | --- | --- | --- | --- | --- | --- | --- | --- | --- | --- | --- | --- | --- | --- |
|  | Replicate 1 | | | | Replicate 2 | | | | Replicate 3 | | | | Summary | |
| Strain | total | # Alive | # Dead | % Alive | total | # Alive | # Dead | % Alive | total | # Alive | # Dead | % Alive | Mean | Std. dev. |
| Control | 41 | 1 | 40 | 2.44% | 35 | 1 | 34 | 2.86% | 54 | 3 | 51 | 5.56% | 3.62% | 1.38% |
| 300 mM Glucose | 47 | 44 | 3 | 93.62% | 52 | 51 | 1 | 98.08% | 45 | 39 | 6 | 86.67% | 92.79% | 4.70% |
| 3 mM MnCl_2_ | 48 | 7 | 41 | 14.58% | 23 | 8 | 15 | 34.78% | 48 | 8 | 40 | 16.67% | 22.01% | 9.07% |
| 100 mM Ascorbate | 34 | 31 | 3 | 91.18% | 27 | 26 | 1 | 96.30% | 39 | 39 | 0 | 100.00% | 95.82% | 3.62% |

Supplementary table 9, cont.

| T-test relative to control | 2 hours | 6 hours | 12 hours |
| --- | --- | --- | --- |
| 300 mM Glucose | 0.00421 | 3.98E-05 | 1.348E-05 |
| 3 mM MnCl_2_ | 0.00932 | 0.000134 | 0.0471057 |
| 100 mM Ascorbate | 0.008972 | 4.6E-05 | 4.638E-06 |

# Supplementary Table 10 – Data and Analysis for Figure 6B

| HBO Exposure Time | 2 hours | | | | | | | | | | | | | |
| --- | --- | --- | --- | --- | --- | --- | --- | --- | --- | --- | --- | --- | --- | --- |
|  | Replicate 1 | | | | Replicate 2 | | | | Replicate 3 | | | | Summary | |
| Strain | total | # Alive | # Dead | % Alive | total | # Alive | # Dead | % Alive | total | # Alive | # Dead | % Alive | Mean | Std. dev. |
| Control | 44 | 32 | 12 | 72.73% | 43 | 37 | 6 | 86.05% | 30 | 18 | 12 | 60.00% | 72.92% | 10.63% |
| 300 mM Glucose | 31 | 31 | 0 | 100.00% | 36 | 36 | 0 | 100.00% | 29 | 28 | 1 | 96.55% | 98.85% | 1.63% |
| 3 mM MnCl_2_ | 36 | 34 | 2 | 94.44% | 34 | 34 | 0 | 100.00% | 36 | 36 | 0 | 100.00% | 98.15% | 2.62% |
| 100 mM Ascorbate | 36 | 36 | 0 | 100.00% | 35 | 34 | 1 | 97.14% | 31 | 29 | 2 | 93.55% | 96.90% | 2.64% |

Supplementary Table 10, cont.

| HBO Exposure Time | 4 hours | | | | | | | | | | | | | |
| --- | --- | --- | --- | --- | --- | --- | --- | --- | --- | --- | --- | --- | --- | --- |
|  | Replicate 1 | | | | Replicate 2 | | | | Replicate 3 | | | | Summary | |
| Strain | total | # Alive | # Dead | % Alive | total | # Alive | # Dead | % Alive | total | # Alive | # Dead | % Alive | Mean | Std. dev. |
| Control | 39 | 17 | 22 | 43.59% | 36 | 5 | 31 | 13.89% | 42 | 19 | 23 | 45.24% | 34.24% | 14.41% |
| 300 mM Glucose | 47 | 46 | 1 | 97.87% | 38 | 36 | 2 | 94.74% | 40 | 39 | 1 | 97.50% | 96.70% | 1.40% |
| 3 mM MnCl_2_ | 35 | 23 | 12 | 65.71% | 42 | 35 | 7 | 83.33% | 35 | 19 | 16 | 54.29% | 67.78% | 11.95% |
| 100 mM Ascorbate | 34 | 29 | 5 | 85.29% | 36 | 26 | 10 | 72.22% | 31 | 25 | 6 | 80.65% | 79.39% | 5.41% |

Supplementary Table 10, cont.

| HBO Exposure Time | 8 hours | | | | | | | | | | | | | |
| --- | --- | --- | --- | --- | --- | --- | --- | --- | --- | --- | --- | --- | --- | --- |
|  | Replicate 1 | | | | Replicate 2 | | | | Replicate 3 | | | | Summary | |
| Strain | total | # Alive | # Dead | % Alive | total | # Alive | # Dead | % Alive | total | # Alive | # Dead | % Alive | Mean | Std. dev. |
| Control | 46 | 3 | 43 | 6.52% | 37 | 1 | 36 | 2.70% | 57 | 2 | 55 | 3.51% | 4.24% | 1.64% |
| 300 mM Glucose | 30 | 21 | 9 | 70.00% | 28 | 17 | 11 | 60.71% | 27 | 13 | 14 | 48.15% | 59.62% | 8.95% |
| 3 mM MnCl_2_ | 42 | 1 | 41 | 2.38% | 33 | 1 | 32 | 3.03% | 42 | 3 | 39 | 7.14% | 4.18% | 2.12% |
| 100 mM Ascorbate | 31 | 17 | 14 | 54.84% | 45 | 25 | 20 | 55.56% | 22 | 14 | 8 | 63.64% | 58.01% | 3.99% |

Supplementary table 10, cont.

| T-test relative to N2 | 2 hours | 4 hours | 8 hours |
| --- | --- | --- | --- |
| 300 mM Glucose | 0.027072 | 0.003646 | 0.0010036 |
| 3 mM MnCl_2_ | 0.031168 | 0.064368 | 0.976319 |
| 100 mM Ascorbate | 0.036429 | 0.014268 | 6.088E-05 |

# Supplementary Table 11 – Data and Analysis for Figure 7A

| 2 °C Cold Shock Duration | 2 hours | | | | | | | | | | | | | |
| --- | --- | --- | --- | --- | --- | --- | --- | --- | --- | --- | --- | --- | --- | --- |
|  | Replicate 1 | | | | Replicate 2 | | | | Replicate 3 | | | | Summary | |
| Strain | total | # Alive | # Dead | % Alive | total | # Alive | # Dead | % Alive | total | # Alive | # Dead | % Alive | Mean | Std. dev. |
| N2 | 48 | 45 | 3 | 93.75% | 69 | 67 | 2 | 97.10% | 50 | 48 | 2 | 96.00% | 95.62% | 1.39% |
| VC289 | 217 | 212 | 5 | 97.70% | 167 | 151 | 16 | 90.42% | 207 | 199 | 8 | 96.14% | 94.75% | 3.13% |
| VC1151 | 26 | 25 | 1 | 96.15% | 16 | 15 | 1 | 93.75% | 29 | 29 | 0 | 100.00% | 96.63% | 2.57% |
| VC754 | 34 | 33 | 1 | 97.06% | 34 | 34 | 0 | 100.00% | 53 | 51 | 2 | 96.23% | 97.76% | 1.62% |
| RB1653 | 34 | 33 | 1 | 97.06% | 24 | 24 | 0 | 100.00% | 36 | 36 | 0 | 100.00% | 99.02% | 1.39% |

Supplementary Table 11, cont.

| 2 °C Cold Shock Duration | 6 hours | | | | | | | | | | | | | |
| --- | --- | --- | --- | --- | --- | --- | --- | --- | --- | --- | --- | --- | --- | --- |
|  | Replicate 1 | | | | Replicate 2 | | | | Replicate 3 | | | | Summary | |
| Strain | total | # Alive | # Dead | % Alive | total | # Alive | # Dead | % Alive | total | # Alive | # Dead | % Alive | Mean | Std. dev. |
| N2 | 74 | 62 | 12 | 83.78% | 52 | 42 | 10 | 80.77% | 52 | 43 | 9 | 82.69% | 82.42% | 1.25% |
| VC289 | 148 | 32 | 116 | 21.62% | 177 | 26 | 151 | 14.69% | 190 | 17 | 173 | 8.95% | 15.09% | 5.18% |
| VC1151 | 23 | 8 | 15 | 34.78% | 36 | 5 | 31 | 13.89% | 31 | 15 | 16 | 48.39% | 32.35% | 14.19% |
| VC754 | 38 | 23 | 15 | 65.00% | 12 | 5 | 7 | 41.67% | 52 | 27 | 25 | 51.92% | 52.86% | 9.55% |
| RB1653 | 36 | 25 | 11 | 69.44% | 31 | 19 | 12 | 61.29% | 44 | 22 | 22 | 50.00% | 60.24% | 7.97% |

Supplementary Table 11, cont.

| 2 °C Cold Shock Duration | 12 hours | | | | | | | | | | | | | |
| --- | --- | --- | --- | --- | --- | --- | --- | --- | --- | --- | --- | --- | --- | --- |
|  | Replicate 1 | | | | Replicate 2 | | | | Replicate 3 | | | | Summary | |
| Strain | total | # Alive | # Dead | % Alive | total | # Alive | # Dead | % Alive | total | # Alive | # Dead | % Alive | Mean | Std. dev. |
| N2 | 39 | 5 | 34 | 12.82% | 66 | 15 | 51 | 22.73% | 33 | 3 | 30 | 9.09% | 14.88% | 5.75% |
| VC289 | 161 | 8 | 153 | 4.97% | 198 | 12 | 186 | 6.06% | 204 | 9 | 195 | 4.41% | 5.15% | 0.68% |
| VC1151 | 30 | 3 | 27 | 10.00% | 27 | 2 | 25 | 7.41% | 26 | 0 | 26 | 0.00% | 5.80% | 4.24% |
| VC754 | 67 | 5 | 62 | 7.46% | 63 | 10 | 53 | 15.87% | 42 | 4 | 38 | 9.52% | 10.95% | 3.58% |
| RB1653 | 38 | 5 | 33 | 13.16% | 50 | 6 | 44 | 12.00% | 34 | 5 | 29 | 14.71% | 13.29% | 1.11% |

Supplementary table 11, cont.

| T-test relative to N2 | 2 hours | 4 hours | 8 hours |
| --- | --- | --- | --- |
| VC289 | 0.73841561 | 5.7682E-05 | 0.076390179 |
| VC1151 | 0.64883112 | 0.007647038 | 0.146857818 |
| VC754 | 0.22879038 | 0.012254232 | 0.458569296 |
| RB1653 | 0.07069484 | 0.017758121 | 0.720441509 |

# Supplementary Table 12 – Data and Analysis for Figure 7A

| HBO Exposure Time | 2 hours | | | | | | | | | | | | | |
| --- | --- | --- | --- | --- | --- | --- | --- | --- | --- | --- | --- | --- | --- | --- |
|  | Replicate 1 | | | | Replicate 2 | | | | Replicate 3 | | | | Summary | |
| Strain | total | # Alive | # Dead | % Alive | total | # Alive | # Dead | % Alive | total | # Alive | # Dead | % Alive | Mean | Std. dev. |
| N2 | 81 | 77 | 4 | 95.06% | 51 | 48 | 3 | 94.12% | 42 | 41 | 1 | 97.62% | 95.60% | 1.48% |
| VC289 | 34 | 10 | 24 | 29.41% | 43 | 23 | 20 | 53.49% | 43 | 15 | 28 | 34.88% | 39.26% | 10.31% |
| VC1151 | 37 | 9 | 28 | 24.32% | 53 | 9 | 44 | 16.98% | 65 | 9 | 56 | 13.85% | 18.38% | 4.39% |
| VC754 | 52 | 50 | 2 | 96.15% | 64 | 61 | 3 | 95.31% | 85 | 79 | 6 | 92.94% | 94.80% | 1.36% |
| RB1653 | 47 | 5 | 42 | 10.64% | 76 | 9 | 67 | 11.84% | 80 | 15 | 65 | 18.75% | 13.74% | 3.57% |

Supplementary Table 12, cont.

| HBO Exposure Time | 4 hours | | | | | | | | | | | | | |
| --- | --- | --- | --- | --- | --- | --- | --- | --- | --- | --- | --- | --- | --- | --- |
|  | Replicate 1 | | | | Replicate 2 | | | | Replicate 3 | | | | Summary | |
| Strain | total | # Alive | # Dead | % Alive | total | # Alive | # Dead | % Alive | total | # Alive | # Dead | % Alive | Mean | Std. dev. |
| N2 | 60 | 24 | 36 | 40.00% | 66 | 29 | 37 | 43.94% | 44 | 17 | 27 | 38.64% | 40.86% | 2.25% |
| VC289 | 75 | 1 | 74 | 1.33% | 80 | 0 | 80 | 0.00% | 66 | 0 | 66 | 0.00% | 0.44% | 0.63% |
| VC1151 | 52 | 1 | 51 | 1.92% | 71 | 0 | 71 | 0.00% | 39 | 0 | 39 | 0.00% | 0.64% | 0.91% |
| VC754 | 72 | 2 | 70 | 2.78% | 62 | 5 | 57 | 8.06% | 59 | 5 | 54 | 8.47% | 6.44% | 2.59% |
| RB1653 | 57 | 1 | 56 | 1.75% | 70 | 1 | 69 | 1.43% | 67 | 1 | 66 | 1.49% | 1.56% | 0.14% |

Supplementary Table 12, cont.

| HBO Exposure Time | 8 hours | | | | | | | | | | | | | |
| --- | --- | --- | --- | --- | --- | --- | --- | --- | --- | --- | --- | --- | --- | --- |
|  | Replicate 1 | | | | Replicate 2 | | | | Replicate 3 | | | | Summary | |
| Strain | total | # Alive | # Dead | % Alive | total | # Alive | # Dead | % Alive | total | # Alive | # Dead | % Alive | Mean | Std. dev. |
| N2 | 51 | 1 | 50 | 1.96% | 84 | 5 | 79 | 5.95% | 64 | 3 | 61 | 4.69% | 4.20% | 1.67% |
| VC289 | 70 | 0 | 70 | 0.00% | 64 | 0 | 64 | 0.00% | 89 | 0 | 89 | 0.00% | 0.00% | 0.00% |
| VC1151 | 70 | 0 | 70 | 0.00% | 77 | 0 | 77 | 0.00% | 53 | 0 | 53 | 0.00% | 0.00% | 0.00% |
| VC754 | 50 | 2 | 48 | 4.00% | 20 | 0 | 20 | 0.00% | 78 | 0 | 78 | 0.00% | 1.33% | 1.89% |
| RB1653 | 51 | 0 | 51 | 0.00% | 70 | 0 | 70 | 0.00% | 66 | 0 | 66 | 0.00% | 0.00% | 0.00% |

Supplementary table 12, cont.

| T-test relative to N2 | 2 hours | 4 hours | 8 hours |
| --- | --- | --- | --- |
| VC289 | 0.00156646 | 1.6521E-05 | 0.02345313 |
| VC1151 | 1.922E-05 | 1.9569E-05 | 0.02345313 |
| VC754 | 0.60481419 | 0.00014365 | 0.18236078 |
| RB1653 | 7.4242E-06 | 1.6022E-05 | 0.02345313 |
